# Specificity-driven graph learning for the identification of rare cell states

**DOI:** 10.1007/s44307-026-00132-9

**Published:** 2026-09-03

**Authors:** Liangzhen Hou, Zheng Hu

**Affiliations:** 1 State Key Laboratory of Quantitative Synthetic Biology, Shenzhen Institutes of Advanced Technology, Shenzhen Institute of Synthetic Biology, Chinese Academy of Sciences, Shenzhen, China; 2 Faculty of Medicine, University of Macau, Macau, Macau SAR, China

## Introduction

Multicellular organisms comprise a diverse array of cell types and cell states, some of which exist at extremely low abundance and appear only transiently within restricted spatial niches or in response to stress and injury. Despite their scarcity, these cells can nonetheless contribute to lineage commitment, tissue regeneration, disease progression, immune evasion, therapy tolerance, and relapse. Single-cell RNA sequencing has provided a high-resolution window for dissecting this heterogeneity, thereby enabling low-abundance populations to be captured in principle (Choi 2019). For cells that have already been captured, the challenge of preserving and reliably identifying rare signals while controlling technical variation remains a central issue in single-cell and spatial transcriptomic analyses. Many existing methods construct neighborhood graphs based on intercellular similarity and propagate information among similar cells. When rare cells lack similar neighbors, their representations are prone to over-smoothing and are ultimately absorbed into high-abundance populations. In multi-sample analyses, batch correction may further weaken signals from rare cell states (Argelaguet et al. 2021; Haghverdi et al. 2018).

## scFormer and the specificity-driven graph strategy

Recently, a team led by Lingling Zheng and Xiao Feng at Sun Yat-sen University published a study in *Advanced Biotechnology* that proposed scFormer, a heterogeneous graph transformer framework designed for the identification of rare cell states (Huang et al. 2026). The central contribution of this work lies in redefining how graph connectivity is established. In this framework, scFormer integrates representation learning, clustering, and optional batch correction into a unified optimization framework. In the primary analysis, the model selects 20 highly specific candidate genes per cell based on Z-scores and constructs bidirectional connections between cells and genes accordingly. Even when rare cells lack similar neighbors, they can establish indirect associations through shared gene nodes, thereby preserving specific expression patterns at the graph construction stage. The heterogeneous graph transformer employs attention mechanisms designed for distinct node types and relation types to learn low-dimensional representations of cells and genes (Ma et al. 2023; Liu et al. 2025; Wang et al. 2024). The clustering component applies discriminative constraints derived from pseudo-labels generated by initial clustering, while also incorporating objectives for expression reconstruction and intra-cluster compactness. For multi-batch datasets, the model can further incorporate losses for global distribution alignment and local structure preservation, thereby mitigating batch effects while seeking to retain rare biological signals. Whereas conventional approaches typically construct a global cell neighborhood before seeking low-abundance populations, scFormer prioritizes gene specificity in establishing connectivity, providing an intuitive rationale for rare-state identification.

## Benchmarking rare-cell identification and batch integration

The study evaluated scFormer and existing approaches (Xu et al. 2024) on simulated data, public single-cell datasets, and multi-batch PBMC data. Across 125 Splatter-simulated datasets (Zappia et al. 2017) generated with an extreme 99:1 imbalance ratio, scFormer achieved a mean F1 score of 0.988 with an F1 variance of 0.008. Across 18 real datasets, reference rare populations were defined as cell types with an abundance of no more than 5% according to the original study annotations. scFormer achieved the highest mean F1 score among the compared methods (0.595), reaching 0.955 on the mouse retina dataset. On a PBMC dataset comprising 30,669 cells across four batches, scFormer achieved an iLISI of 2.695, exceeding that of Harmony at 2.191 (Korsunsky et al. 2019). The F1 score, precision, and recall for rare-cell identification were 0.675, 0.977, and 0.516, respectively (Wegmann et al. 2019). Collectively, these results demonstrate that scFormer delivers stable performance in rare-cell identification across complex, imbalanced datasets and multi-batch data.

## Recovery of rare epithelial and intestinal populations

Beyond quantitative benchmarks, the authors further examined scFormer’s ability to recover known populations and candidate states in real data. In the mouse airway epithelium dataset, scFormer detected *Foxi1*-positive pulmonary ionocytes and goblet cells, and identified two candidate states associated with basal cells and club cells, respectively, both exhibiting proliferative features (Montoro et al. 2018). These populations remained partially detectable after removing a set of cell-cycle-related genes. In the mouse intestinal crypt regeneration dataset, the model identified *Clu*- and *Anxa1*-expressing revival stem cells (revSCs) (Ayyaz et al. 2019), as well as low-abundance immune subpopulations with B-cell and plasmacytoid dendritic cell characteristics that were detected almost exclusively in unirradiated samples. These subpopulations exhibited compositional differences associated with tissue injury status.

## Rare cell states in spatial transcriptomics

In the representative slide_4E section of the sci-Space mouse embryo spatial transcriptomics dataset (Srivatsan et al. 2021), scFormer mapped a biliary-epithelial-like state (Amarachintha et al. 2022) at 0.92% abundance and a pancreatic-marker-enriched state at 0.52% abundance to their original spatial coordinates. The two states exhibited spatial self-enrichment of 35.74-fold and 89.52-fold, respectively, relative to a permuted background, indicating pronounced spatial aggregation within that section. These findings demonstrate that scFormer can identify low-abundance populations from both single-cell and spatial transcriptomic data.

## Conceptual significance and future prospects

The significance of scFormer extends beyond accurately identifying rare cell states. By allowing highly specific genes to participate in defining connectivity, the method reduces dependence on high-abundance cell neighborhoods and offers a promising approach to rare-state identification in multimodal atlases, perturbation screens, and larger-scale tissue datasets. The specificity-driven graph construction strategy offers a new connectivity strategy for rare-cell-state analysis. As single-cell and spatial atlases continue to grow, ensuring that such low-abundance yet biologically meaningful states remain detectable will become increasingly important. scFormer represents a viable starting point in this regard, and its subsequent validation across multimodal data, perturbation screens, and clinical samples is expected to further test and extend the value of this approach.
